# Bibliographical

**Published:** 1856-12

**Authors:** 


### BIBLIOGRAPHICAL.

We have received from the Publishers, Messrs. Blanchard & Lea, of Philadelphia, "The Practical Anatomist; or, The Student's Guide, in the Dissecting-Room. By I. M. Allen, M. D., Late Professor of Anatomy in the Medical Department of Pennsylvania College, &c., &c." This is a work of over 600 pages, with 266 illustrations, the character of which is clearly indicated by its title. From an examination of the work we have no doubt that this is one of the most valuable aids that we have had furnished for the study of Practical Anatomy. The Author remarks that "In aiming to make the book as useful as possible, viewing the Student as a candidate for the Practice of Medicine and Surgery, I have not hesitated to discriminate, to some extent, between different regions and organs in a practical point of view. Hence I have dwelt longer on some points than on others, being governed in this respect partly, by what I conceived to be the relative value and importance of any part or organ, to the Student, and partly by the difficulty which I have observed Students, in the dissecting room, especially beginners, to have in dissecting and understanding them. Thus I have devoted a large share of time and space to the organs contained in the three great splanchinic cavities, to the organs of the special senses, and to such regions as the perineum, the inguinal, the femoral, the anterior part of the neck and the axilla.

Although the space allowed in the original plan of the book, did not admit of my dwelling long on the Medical and Surgical Anatomy, of many parts and regions, I have endeavored to direct the attention of the Student to whatever had a practical bearing, so that he could, by referring to works on Medicine and Surgery, derive full advantage from his dissections. To give merely a *meagre* or *superficial* account of the Medical and Surgical Anatomy of a part, I am satisfied does the Student more harm than good. The work will be found to be valuable, not only to the student in the dissecting-room, but will furnish valuable aid to the physician as a book of reference, in refreshing his memory as to the position and relation of the different parts of the body."

We have received, also, from Messrs. A. S. Barnes & Co., 51 and 53, John Street, New York, the "Hand-book of In-organic Chemistry for the use of Students. By William Gregory, M. D., F. R. S. E., Professor of Chemistry in the University of Edinburgh and Author of Hand-book of Organic Chemistry." To which, is added, The Physics of Chemistry. By J. Milton Sanders, M. D., L. L. D., Professor of Chemistry in the Eclectic Medical Institute of Cincinnati.

The work seems to have been well gotten up by the publishers, and may be a valuable book, but we must confess that our repugnance to everything bearing the name of *Modern Eclecticism*, is such, that we have had no disposition to look beyond the title page and preface—we have no taste for, or confidence in, the writings of those whose minds are either infatuated by any of the species of quackery, or who, for dishonorable ends, are sailing under false colors; men of this class, we think, should not be trusted, even in the exact sciences. To the publishers we are obliged for the courtesy extended in sending the work, and we are only sorry that they have not sent us a book that we could have aided in circulating.

Since the above was written, we have received "The Hand-book of Organic Chemistry," By the same Author and Editor, from the same publishers, Messrs. A. S. Barnes & Co., 51 and 53, John Street, New York; to which the same remarks apply.

We also find upon our table the Annual Circular of the Medical Department of the University of Louisiana, for the Session of 1856–7. This Institution is located in New Orleans, and has been in successful operation for a number of years. The regular Annual Course of Lectures, commenced on Monday the 17th of November, and will terminate in March, 1857. We learn from the Circular that there were 222 Matriculants, and 65 Graduates in 1855–6, and that the Students of the Class were from Louisiana, Mississippi, Alabama, Texas, Arkansas, Tennessee, Kentucky, Iowa, Missouri, South Carolina, Georgia, Florida, Indiana and France. The Faculty announces to the public the continued prosperity of the Medical College of Louisiana. More than twenty-three hundred names are on the Register of Matriculants. The College is endowed by the State.

